# Effects of *Helicobacter pylori* Infection on the Development of Chronic Gastritis

**DOI:** 10.5152/tjg.2023.22316

**Published:** 2023-07-01

**Authors:** Xiaoming Zhou, Yongming Zhu, Jiayu Liu, Jindi Liu

**Affiliations:** 1 Nantong Hospital to Nanjing University of Chinese Medicine, Nantong, Jiangsu, China

**Keywords:** Chronic gastritis, *Helicobacter pylori*, gene, RNA

## Abstract

**Background/Aims:**

Based on the gene expression profiles of gastric epithelial tissue at different stages of *Helicobacter pylori*-infected gastritis, key long noncoding RNAs and genes in the development of *Helicobacter pylori* infection-induced gastritis were screened to provide a basis for early diagnosis and treatment.

**Materials and Methods:**

We downloaded 2 sets of sample data from the database, including gastric epithelial tissue samples from gastritis patients from Bhutan and Dominican, and screened mRNAs in the differentially expressed RNAs of the 2 regions. Mfuzz clustering algorithm was used to screen RNAs related to the 3 stages of chronic gastritis. The competing endogenous RNA (ceRNA) regulation network was constructed, and the selected key RNAs were verified. Samples from Bhutan and Dominican were subdivided into the chronic gastritis/normal comparison groups, and the differentially expressed RNAs were screened to obtain 1067 overlapping RNAs, containing 21 long noncoding RNAs and 1046 mRNAs.

**Results:**

Thirty-eight significant gene ontology functional nodes and 6 expression pattern clusters were obtained. Two ceRNA regulatory networks were constructed, and 4 shared miRNAs (hsa-miR-320b, hsa-miR-320c, hsa-miR-320d, and hsa-miR-155-5p) were obtained. Eleven important long noncoding RNAs (AFAP1-AS1, MIR155HG, LINC00472, and FAM201A) and mRNAs (CASP10, SLC26A2, TRIB1, BMP2K, SCAMP1, TNKS1BP1, and MBOAT2) regulated by these 4 miRNAs were obtained. These results indicated that *Helicobacter pylori* infection had a certain influence on the development of gastritis.

**Conclusion:**

The 11 key RNAs can provide a target for the early diagnosis and treatment of chronic gastritis following *Helicobacter pylori* infection.

**Main Points**

- Mfuzz clustering algorithm was used to screen RNAs related to the 3 stages of chronic gastritis (CG).
- Two ceRNA regulation networks were constructed, and 4 shared miRNAs were obtained.
- The 11 key RNAs screened can provide targets for early diagnosis and treatment of CG development after *Helicobacter pylori* infection.

## INTRODUCTION

Chronic gastritis (CG) is a common and frequently occurring disease of the digestive system. It is a disease of the intrinsic gland of the mucosa caused by repeated damage to the gastric mucosa. Chronic gastritis is nearly associated with the occurrence of gastric cancer (GC),^1^ which is one of the most common malignant tumors. The mortality rate is third only to lung cancer and liver cancer, and the survival rate is only approximately 20% within 5 years, which is a serious threat to human survival and health.^2,3^

*Helicobacter pylori* (Hp) is a unipolar, multiflagellate, blunt-ended, spiral-curved, microaerobic, Gram-negative bacterium with a length of 2.5-4.0 μm and a width of 0.5-1.0 μm.^4^ *Helicobacter pylori* has strong exercise capacity, can penetrate the mucus layer to damage gastric mucosal epithelial cells, and is a major pathogen that can survive in the human stomach for a long time, which is related to the development of CG and peptic ulcer.^5^ *Helicobacter pylori* infection occurs mainly through 3 stages: (1) stable colonization in gastric mucosal epithelial cells; (2) the evasion from host immune system attack; and (3) the release of toxins that damage the gastric mucosa.^6-8^ *Helicobacter pylori* infection can trigger innate and adaptive immune responses of the host, resulting in the infiltration of several neutrophils, monocytes, and macrophages, leading to the occurrence of acute gastritis and CG. *Helicobacter pylori* induces the host to produce various cytokines that alter the physiological environment in the stomach.^9-11^

Recently, studies have found that the abnormal expression of long noncoding RNAs (lncRNAs) at the cellular level is closely associated with the occurrence and development of cancers. Long noncoding RNAs participate in various biological behaviors, such as cell differentiation and ontogeny, at multiple levels. However, information about the function of most ncRNAs is limited; so they have broad research prospects.^12,13^ Long noncoding RNAs are a class of ncRNAs that are greater than 200 nt in length and lack the ability to encode proteins. Compared with RNA-encoding proteins, lncRNAs are shorter in length, have fewer exons, perform less coding, and have tissue or cell specificity.^14^

Existing studies have confirmed that the occurrence of diseases is often associated with abnormal transcription. This abnormality not only is limited to abnormalities in protein-encoding RNA levels but also includes abnormalities in the function of ncRNAs in the genome, including lncRNAs. Long noncoding RNAs are noncoding RNAs that play an important regulatory role in the development of some diseases and may serve as a biomarker for disease progression or prognosis. Researchers have discovered that many functional lncRNAs can directly or indirectly regulate the expression of known oncogenes or tumor-suppressor genes, indicating that lncRNAs have broad prospects for research and development as a tool for tumor diagnosis and treatment.^15,16^

## MATERIALS AND METHODS

### Grouping and Preprocessing of Experimental Data

On March 14, 2019, we downloaded 32 samples of the data number GSE60427 from the National Center for Biotechnology Information Gene Expression Omnibus database (https://www.ncbi.nlm.nih.gov/geo/).^17^ These 32 samples, which are stomach epithelial tissue samples from patients with gastritis from Bhutan and Dominican, contain gastric epithelial tissue obtained from healthy individuals and those with mild, severe, and intestinal metaplasia (IM) *Helicobacter pylori*-infected gastritis. The detection platform used was GPL17077: Agilent-039494 SurePrint G3 Human GE v2 8x60K Microarray 039381. Additionally, a set of GST111762 gene expression profile data of gastric epithelial cells was downloaded, including 15 samples (i.e., 3 healthy samples, 6 Hp-infected gastritis samples, and 6 Hp-infected CG samples), and the detection platform used was GPL15314Arraystar Human lncRNA microarray V2.0. This dataset served as a secondary validation factor dataset for post-analysis.

The original expression level data of the 2 datasets were downloaded in the original format of TXT, and then, the expression spectrum data were log_2_ logarithmized using the Limma package version 3.34.0 in the R3.4.1 language (https://bioconductor.org/packages/release/bioc/html/limma.html)^18^ so that the gene expression data were converted from a skewed distribution to approximately normal distribution, and then, the data were normalized using the median normalization method.

### Significant Differential Expression of RNA

First, the annotation platform of GSE60427 was downloaded, and the detection sequence provided by the platform was used to align Clustal 2 (http://www.clustal.org/clustal2/)^19^ using human whole-genome sequencing (version: GRCh38) to identify lncRNAs and mRNAs and their corresponding expression information.^20,21^

Since the samples were from 2 regions, to eliminate regional differences, we first classified the samples according to the presence or absence of gastritis in the Bhutan and Dominican samples. Then, the Limma package version 3.34.0 in the R3.4.1 language^18^ was used to calculate the differential false discovery rate and fold-change value of RNA expression between the groups. False discovery rates less than 0.05 and |log2FC| more than 0.5 were regarded as the threshold for screening the differentially expressed RNAs (DERs). Based on the expression level of the RNAs obtained by screening, the expression values were subjected to bidirectional hierarchical clustering based on the Euclidean distance^22,23^ using the Pheatmap package version 1.0.8 (https://cran.r-project.org/package = pheatmap)^24^ in R3.4.1 language and displayed using a heat map.

The DERs obtained in the Bhutan and Dominican regions were compared, and then, the intersection of the 2 was considered a set of DERs after eliminating geographical differences. The DAVID 6.8-based (https://david.ncifcrf.gov/)^25,26^ gene ontology (GO) functional node enrichment annotation analysis was performed on the mRNA in the DERs, and the significantly associated GO functional nodes and KEGG (Kyoto Encyclopedia of Genes and Genomes) signaling pathways were screened, and *P* values of less than .05 were selected as the significant enrichment screening threshold.

### Mfuzz Clustering Algorithm for Screening RNAs Related to Different Stages of Chronic Gastritis

In the analysis, the CG samples contained disease samples at different stages of development—mild, severe, and IM stages—which are the process of deteriorating from mild to severe during the development of CG, and screening of DERs that express regular changes with stage changes in DERs can help study and screen RNAs closely related to disease progression. We used the Mfuzz package version 2.42.0 (http://www.bioconductor.org/packages/release/bioc/html/Mfuzz.html) in the R3.4.1 language^27^ to perform time-series trend analysis on DERs according to the CG level and obtain the expression trend module gene clustering. The expression trends of differential genes during CG deterioration were observed, and then, the significant module gene sets were analyzed using the DAVID 6.8-based^25,26^ GO biological process and KEGG signaling pathway enrichment annotation.

### Construction of ceRNA Regulatory Networks

According to the membership value of RNAs in the trend module obtained by Mfuzz clustering in the previous step, key RNAs were identified in each module, and then, the ceRNA (competing endogenous RNA) network was constructed as follows:

- Prediction of lncRNA–miRNA linkage relationship: For the lncRNAs, the binding relationship between the target lncRNAs and miRNAs was searched using the DIANA-LncBasev2 database information (http://carolina.imis.athena-innovation.gr/diana_tools/web/index.php?r=lncbasev2%2Findex-experimental),^28^ and the linkage of miRNA target gene (miTG) score was higher than 0.8. The miTG-score is defined as the sum of the scores of all identifiable microRNA response elements at the 3ʹ-UTR. The higher the value, the greater the probability of targeting.
- Prediction of miRNA–mRNA linkage relationships: For the miRNAs obtained in A, the target genes regulated by them were searched using the starBase version 2.0 database.^29^ The starBase database provides comprehensive target gene prediction information from TargetScan, PicTar, RNA22, PITA, and miRanda. We screened at least one of the database’s regulation relationships, which were considered target miRNAs to regulate the target gene’s relationship pairs and corresponded to the mRNA in the key RNA set, retaining the key target mRNA ligation pairs regulated by miRNA.
- Construction of the ceRNA network: The linkages were combined between A and B; then, a lncRNA–miRNA–mRNA regulation network was constructed, and the DAVID-based GO biological processes and KEGG signaling pathway enrichment analysis were performed for each ceRNA to regulate mRNA in that network.

### Verification of the Expression Levels of Key RNAs

The various ceRNA regulatory networks constructed in the fourth step were compared, and different ceRNA networks regulated by the same miRNAs were selected to integrate the key RNAs in different modules. For the selected key RNAs, we first showed the expression level of CG/normal in the original analysis dataset GSE60427 and then showed the curve of the expression level changes in different developmental stages of CG. Additionally, key RNAs were validated in healthy controls and CG samples in GSE111762, and the expression levels were further validated at different stages of disease progression.

## RESULTS

### Data Preprocessing and Significant Differential Expression Screening

**Figure 1.**
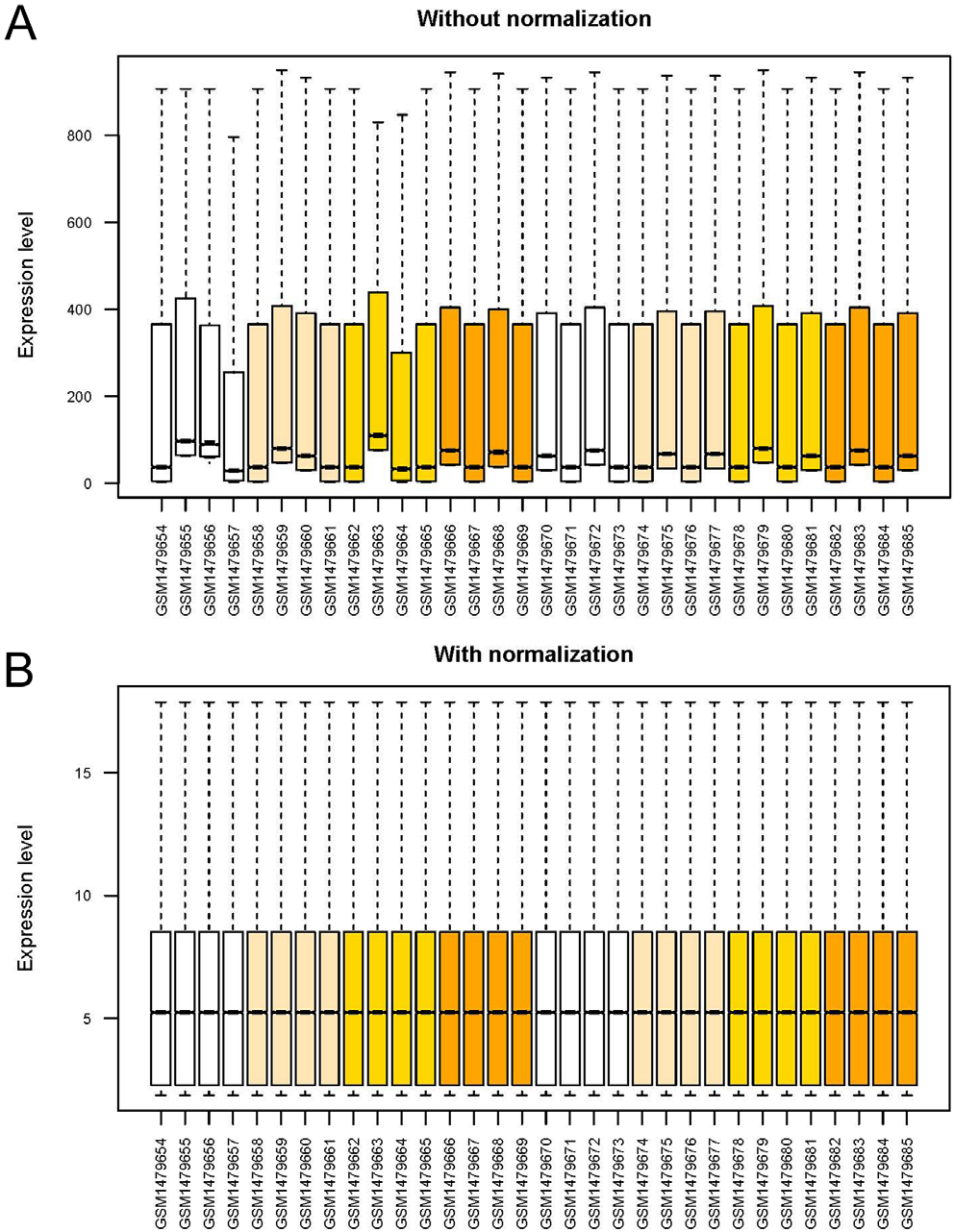
The box diagram of GSE60427 before (A) and after (B) standardization.

**Figure 2.**
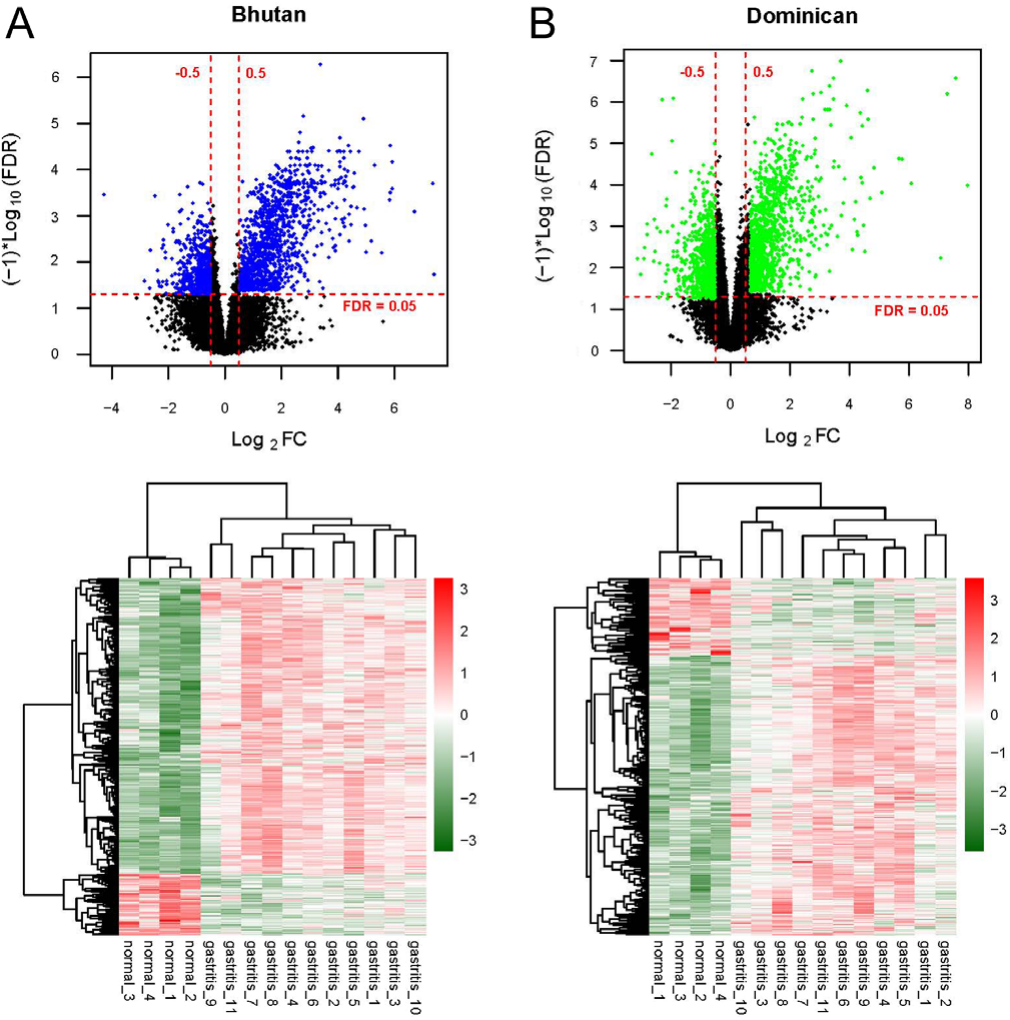
The volcano map of Bhutan (A) and Dominican (B) areas (above). The blue and green dots indicate differentially expressed RNAs (DERs) in the comparison group of the 2 regions, the black dots indicate non-DERs, the red horizontal dashed lines indicate false discovery rates of less than 0.05, and the 2 red vertical dashed lines indicate |Log2FC| more than 0.5.

First, the data of the expression spectrum dataset obtained by downloading were standardized. The box diagram before and after standardization is shown in Figure 1. The samples were then divided into Bhutan and Dominican samples according to the different regions from which the samples were obtained. The samples were further subdivided into the CG/normal comparison groups. The DERs of the CG/normal comparison group of the 2 regions were screened using the Limma package; 1859 (i.e., 1789 mRNAs and 70 lncRNAs; 633 downregulated and 1226 upregulated expressions) and 2098 (i.e., 2047 mRNA and 51 lncRNA; 764 downregulated and 1334 upregulated expressions) DERs were screened. The volcano map is shown in Figure 2A and 2B. The bidirectional hierarchical clustering heat map based on the 2 DER expression levels obtained by screening is shown in Figure 2A and 2B. It can be seen from the figure that the RNA expression values obtained by screening can separate the different types of samples well, and the color is clear, indicating that the RNAs screened in the CG and control groups in the 2 regional groups are characteristic of the samples.

### Comparative Analysis of Differentially Expressed RNAs Collected from Different Regions

**Figure 3.**
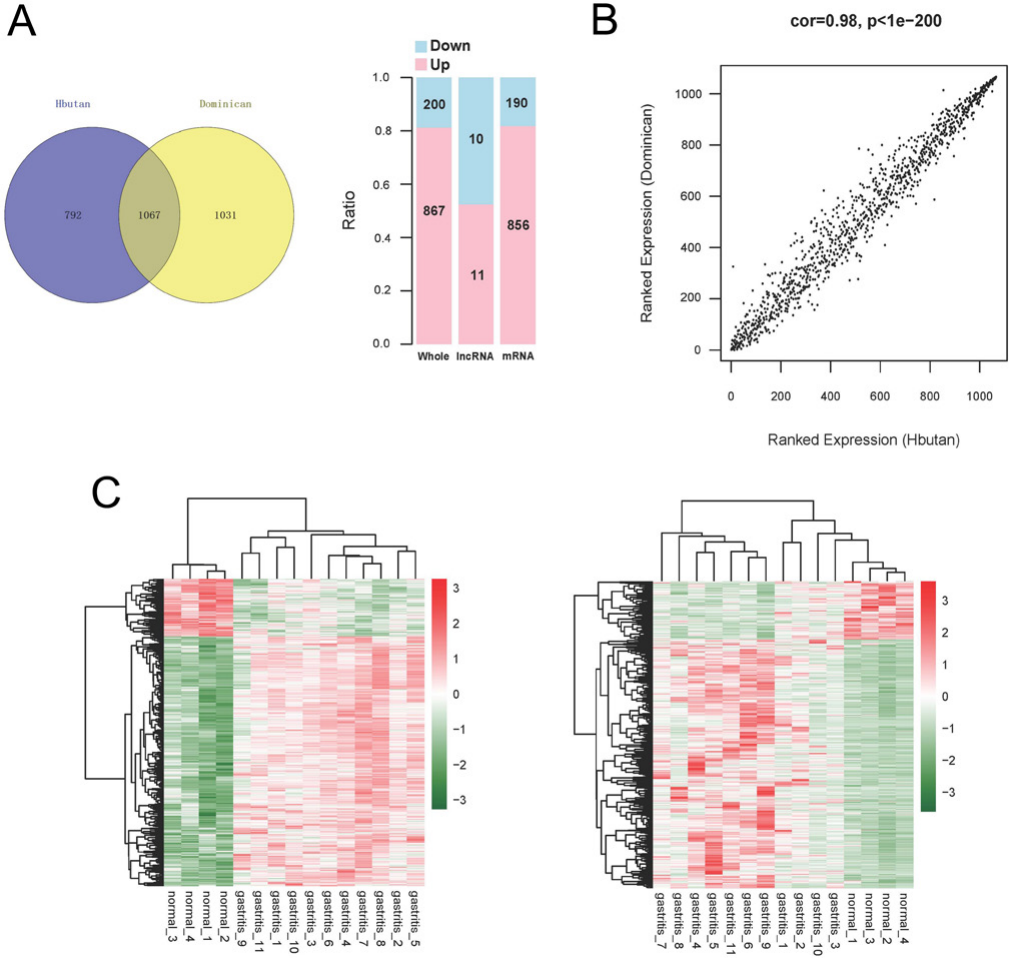
In the Bhutan and Dominican regions, the collected differentially expressed RNAs of the Venturi and overlapping genes up-and-down proportional distribution histograms (A). Scatterplot of the correlation of overlapping gene expression levels in the Bhutan and Dominican regions (B). Bidirectional hierarchical clustering map of overlapping levels of expression in the 2 comparison groups (C).

The DERs screened in the Bhutan and Dominican samples were then compared, and the results are shown in Figure 3A. Overall, 1067 overlapping RNAs were obtained, of which 200 were downregulated and 867 were upregulated to express RNAs, including 21 lncRNAs and 1046 mRNAs. The distribution ratio is shown in Figure 3A. Then, we analyzed the correlation between the expression levels of overlapping RNAs in disease samples from the Bhutan and Dominican regions, and the results are shown in Figure 3B. The results showed that overlapping RNAs, although from CG samples from different regions, showed a high positive correlation. Additionally, based on overlapping RNAs, bidirectional hierarchical clustering analysis based on the expression levels was performed, and the results are shown in Figure 3C. The results showed that the degree of difference between the overlapping RNAs in the 2 groups was highly significant, and the direction of the difference was the same. In summary, the difference between the samples from different regions was eliminated by taking the intersection of DERs obtained from the 2 regions (helping combine different regional samples for different stages of disease development).

**Figure 4.**
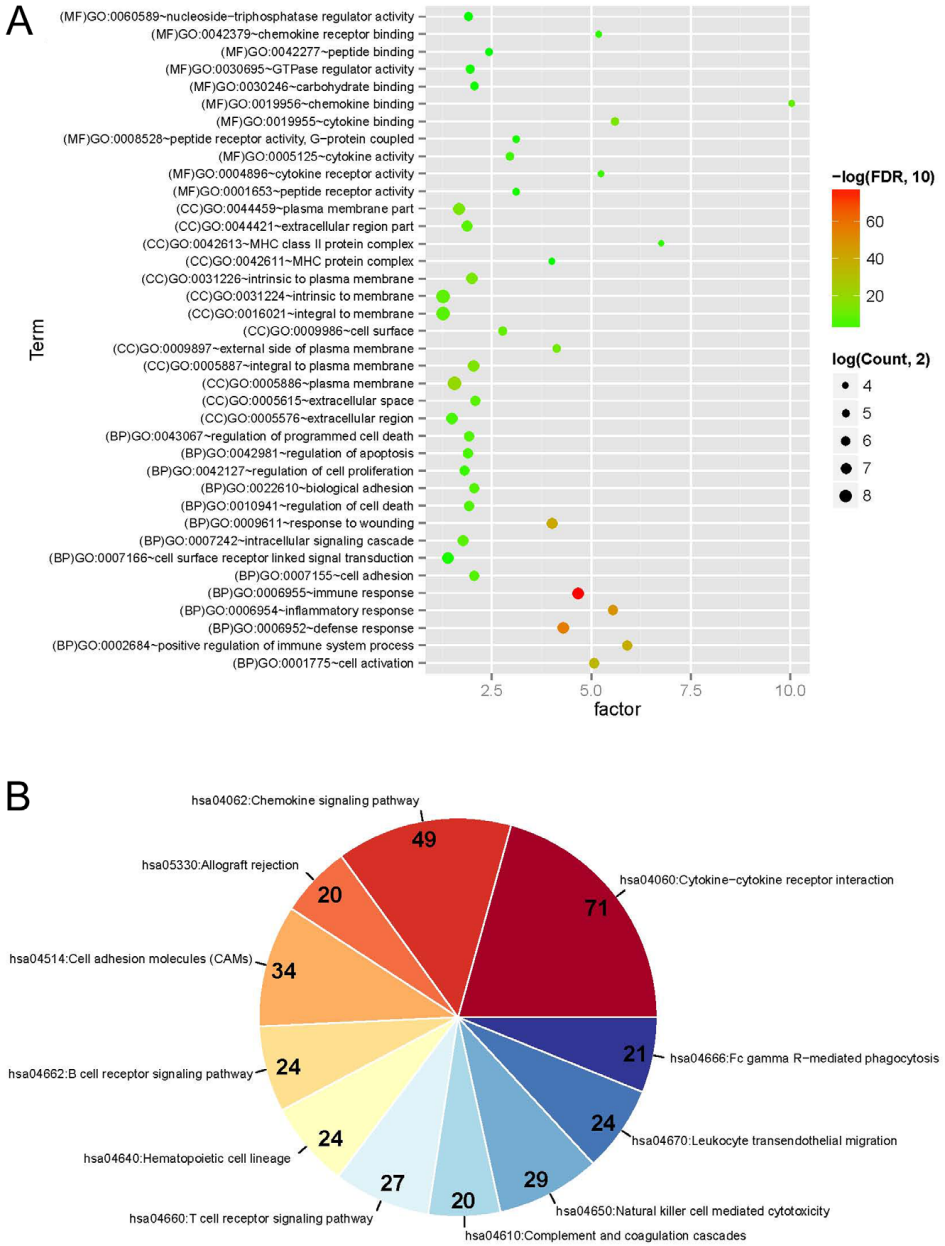
(A) Bubble point map of gene ontology function nodes with significant enrichment of overlapping genes. The horizontal axis represents rich factors, and the vertical axis represents the name of the item. The size of the point represents the number of genes involved. The larger the point, the more the number of genes; the color of the dots represents the correlation, and the closer the color is to red, the higher the significance. (B) KEGG signaling pathway pie chart with significant enrichment of overlapping genes. Each component represents a different KEGG pathway, the number represents the genes number involved in the pathway, the color represents significance, and the closer to red, the higher the significance.

Then, the mRNAs contained in the overlapping RNAs were subjected to enrichment annotation analysis based on the DAVID-based GO functional node and KEGG signaling pathway. The results are shown in Table 1 and Figure 4. Thirty-eight significantly related GO functional nodes were obtained, including 14 biological processes, 13 cellular components, and 11 molecular functions. Overlapping genes were significantly involved in biological processes, such as immune responses, and in 11 KEGG signaling pathways, including cytokine–cytokine receptor interactions.

### Mfuzz Clustering Algorithm to Screen RNAs Related to Different Stages of Chronic Gastritis

**Figure 5.**
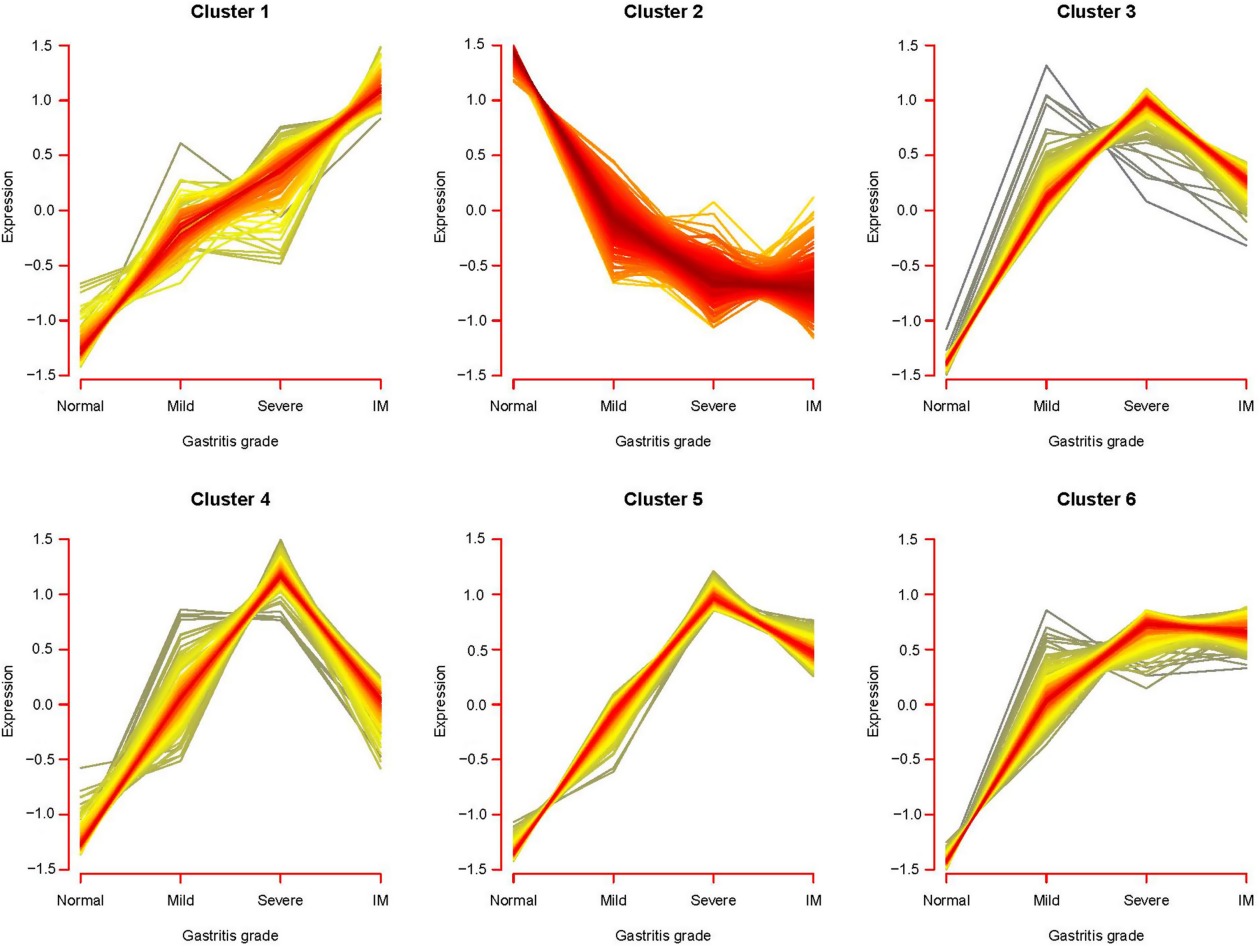
Overlapping mRNAs are based on the expression pattern clustering map of the Mfuzz algorithm.

**Figure 6.**
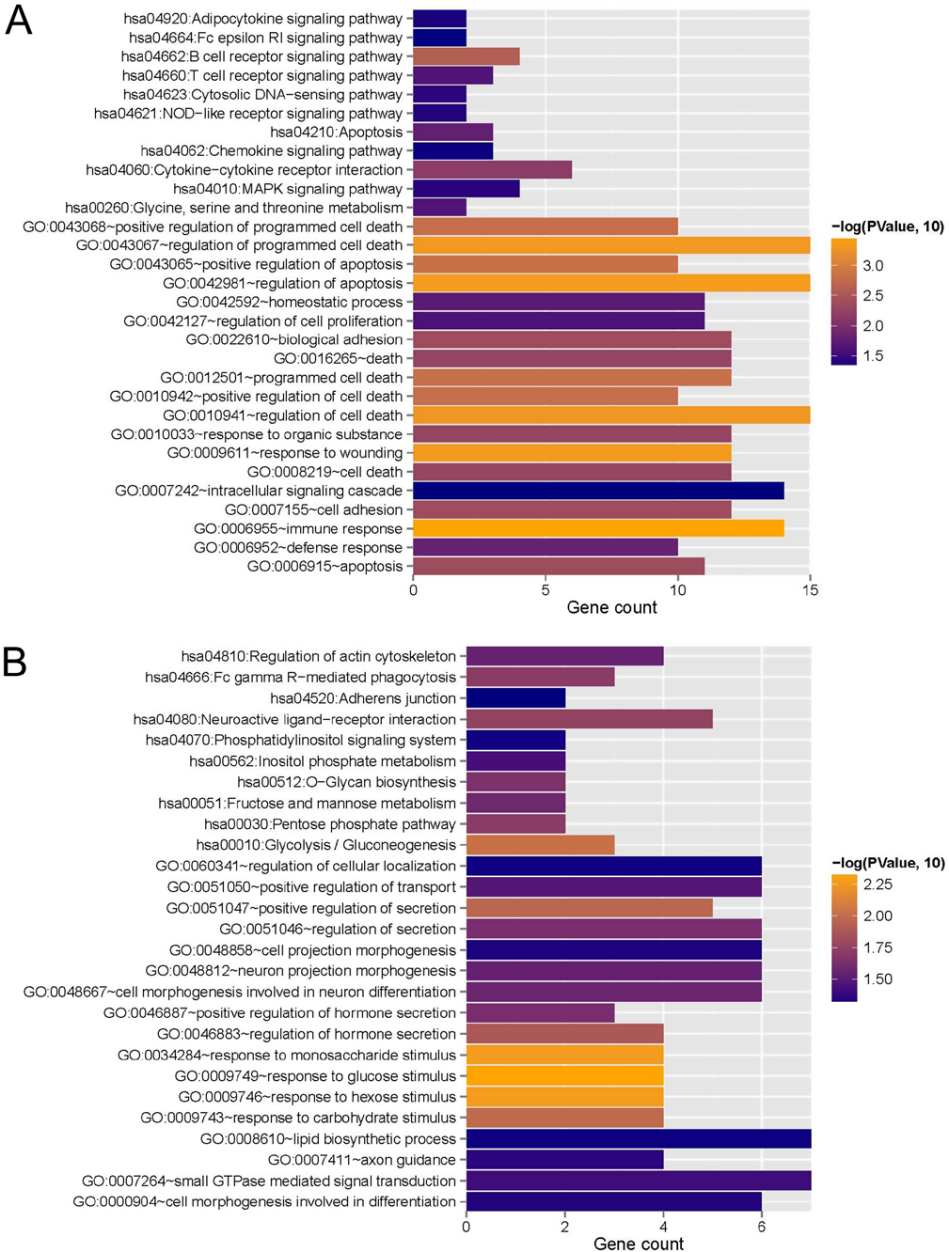
The mRNAs contained in clusters 1 (A) and 2 (B) are significantly correlated with the gene ontology biological process and KEGG signaling pathway. The horizontal axis indicates the gene number, the vertical axis indicates the item name, the color of the column represents significance, and the closer the color is to orange, the higher the significance.

In the sample analysis, the CG samples contained disease tissues at different stages of development—mild, severe, and IM stages—which comprise the process of deterioration from light to severe in the development of CG. Screening for DERs in regular overlapping DERs with regular changes in expression of stages can help examine and screen RNAs closely related to disease progression. We used the Mfuzz package to analyze the time-series trend of the overlapping DERs according to the CG level. The results are shown in Figure 5; 6 expression pattern clusters were obtained. Among them, the genes contained in clusters 1 and 2 maintained a single change state during the normal–mild–severe–IM deterioration development stage, and the expression continued to rise and fall. Therefore, we speculated that the continuous changes in gene expression in these 2 modules are closely related to the progression of CG. The enrichment annotation analysis of GO biology and KEGG signaling pathways was performed on mRNAs contained in clusters 1 and 2, and the results are shown in Tables 2 and 3 and Figure 6.

### Construction of the ceRNA Regulatory Network

**Figure 7.**
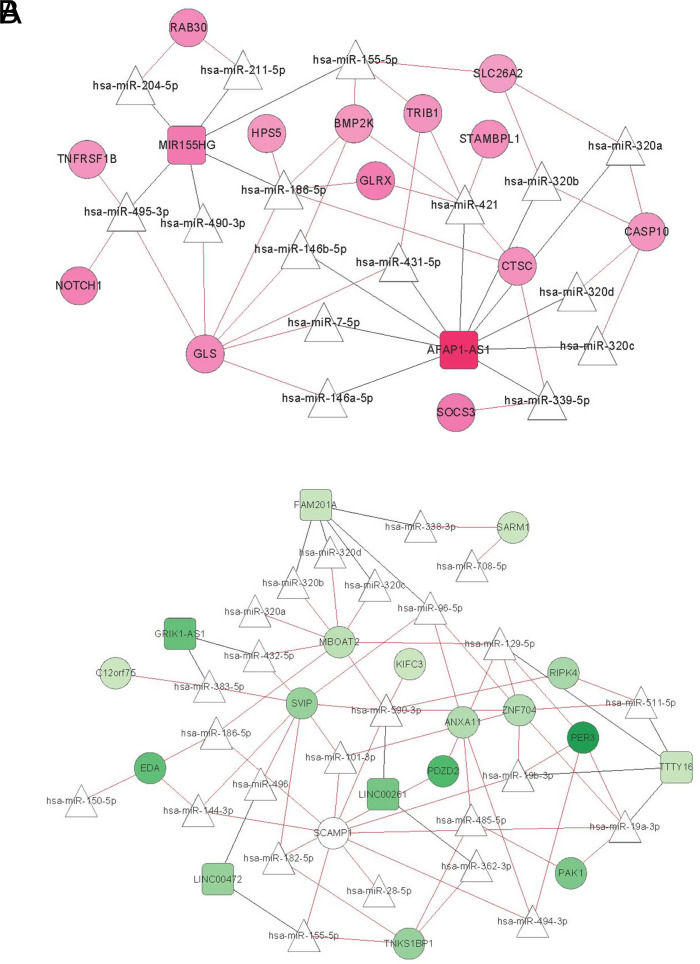
The competitive endogenous RNA regulatory network of RNAs in clusters 1 (A) and 2 (B). Square and circle indicate lncRNA and mRNA, respectively; triangle indicates miRNA; and black and red connections indicate lncRNA–miRNA and miRNA–mRNA connection relationships, respectively.

According to the membership value of the RNAs in the trend module obtained by Mfuzz clustering in the previous step, the gene membership values in clusters 1 and 2 were sorted by power. RNAs with a membership value higher than 0.6 were selected as key genes in clusters 1 and 2, containing 30 RNAs (i.e., 2 lncRNAs and 28 mRNAs) and 190 RNAs (i.e., 8 lncRNAs and 182 mRNAs), respectively. Then, the following analysis was performed:

- Long noncoding RNA–miRNA connection relationship prediction: For the 2 and 8 lncRNAs contained in clusters 1 and 2, respectively, the binding relationship between the target lncRNA and miRNA was searched using DIANA-LncBasev2 database information. Only the ligation of miTG scores above 0.8 was retained; 16 and 27 pairs of lncRNA–miRNA connections in clusters 1 and 2, respectively, were screened.
- miRNA–mRNA connection relationship prediction: For the miRNAs obtained in A, the target genes regulated by them were searched using the starBase version 2.0 database. Then, the target gene is mapped into the mRNAs contained in the 2 clusters; 33 and 56 pairs of miRNA–mRNA regulation connections of miRNAs linked to clusters 1 and 2 lncRNAs, respectively, in step A were obtained.
- Construction of a ceRNA regulatory network: Combining the regulation relationships in A and B, a ceRNA regulatory network composed of RNAs in clusters 1 and 2 was constructed (Figure 7). The GO biological process and KEGG signaling pathway were then performed on the mRNAs in the 2 ceRNA regulatory networks (Tables 4 and 5). Screening yielded 6 and 6 significant correlations in GO biological processes and 1 and 0 KEGG signaling pathways, respectively.

### Verification of the Expression Levels of Key RNAs

**Figure 8.**
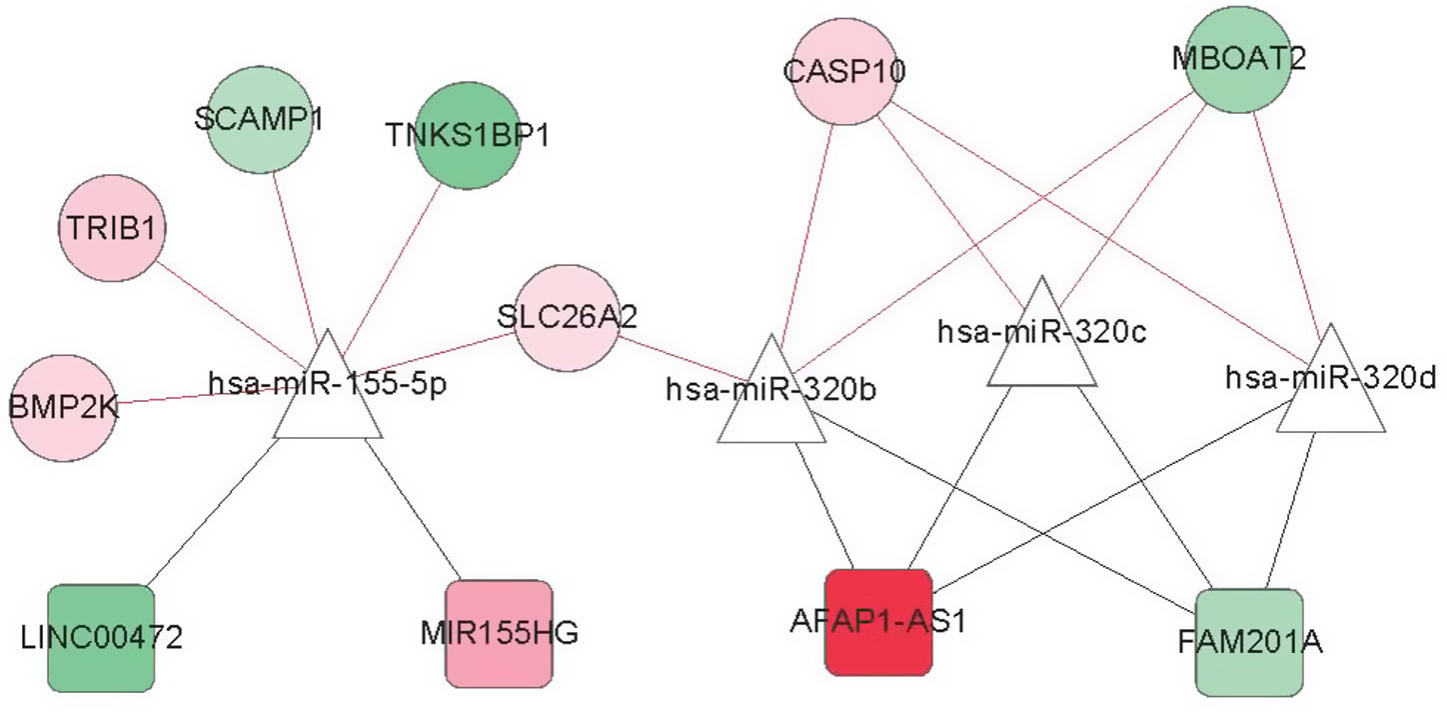
The competitive endogenous RNA regulatory network regulated by consensus miRNAs. Square and circle indicate lncRNA and mRNA, respectively; triangle indicates miRNA; black and red connections indicate lncRNA–miRNA and miRNA–mRNA connection relationships.

Comparing the 2 ceRNA regulatory networks constructed in the fourth step, 4 shared miRNAs were obtained: hsa-miR-320b, hsa-miR-320c, hsa-miR-320d, and hsa-miR-155-5p. The ceRNA regulatory network involved in these 4 miRNAs was extracted (Figure 8). Eleven important lncRNAs and mRNAs regulated by these 4 miRNAs were screened: 6 in cluster 1 (i.e., AFAP1-AS1, MIR155HG, TRIB1, BMP2K, SLC26A2, and CASP10) and 5 in cluster 2 (i.e., LINC00472, FAM201A, SCAMP1, TNKS1BP1, and MBOAT2).

**Figure 9.**
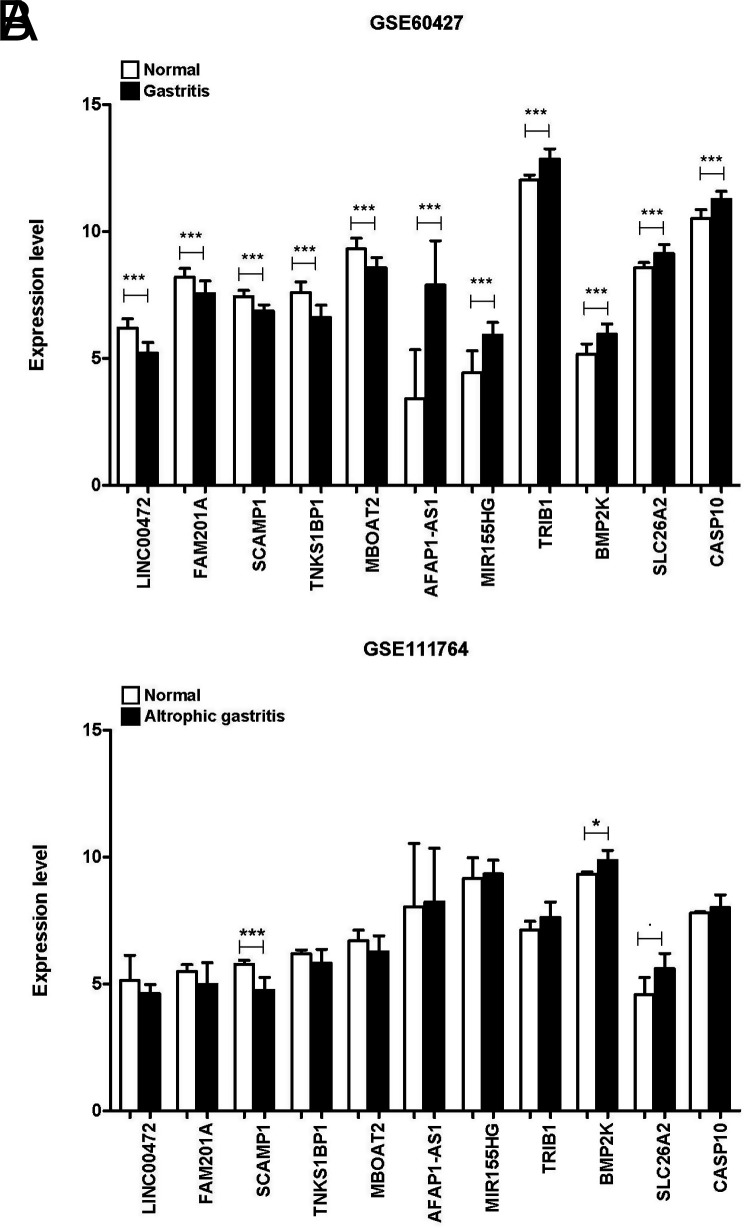
Expression levels of the 11 RNAs in chronic gastritis samples: healthy control samples in GSE60427 (A) and GSE111764 (B).

For the 11 key RNAs selected, we first showed the CG/normal expression level in the original analysis dataset GSE60427 (Figure 9A). Additionally, expression levels were verified for the 11 key RNAs in 3 healthy control samples and 6 CG samples in GSE111762 (Figure 9B). The expression levels of the 11 RNAs in different samples of the 2 datasets were the same; SCAMP1 and BMP2K were significantly different between the 2 sets of healthy control and CG samples.

## DISCUSSION

Global cancer statistics show that GC ranks second in cancer incidence in developing countries and third in mortality. Approximately 70% of new cases and deaths each year come from developing countries.^2,3^ Since the discovery of Hp in 1984, which has led to stomach and duodenal ulcers, Hp has been studied more deeply as the initiator of gastritis. Until 1991, Knight et al^30-33^ have found that Hp infection can increase the risk of stomach cancer, and the risk is almost 3 times higher than that of people without Hp infection.

Several studies have reported that Hp infection of host cells causes instability of the genome inside the cell.^34,35^ In numerous malignant tumors, treatment is more difficult because of their abnormal length and rate of migration. If we can find the target genes that may regulate the growth of tumors or distant metastasis from the terminal based on molecular biology, it will undoubtedly bring hope to the treatment of tumors. Numerous studies have shown that the abnormal expression of various inflammatory cytokines may increase the risk of GC.^36^

Chang et al^37^ summarize the evidences of Hp virulence factors in relation with gastroduodenal diseases, and infection with Hp-carrying specific virulence factors is associated with increased risk of serious clinical outcomes. The study by Chang et al^38^ confirmed that 3 miRNAs (miR99b-3p, miR-564, and miR-638) were significantly higher in 3 Hp-positive cancer tissues than in Hp-negative cancer tissues. Furthermore, 4 miRNAs (miR-204-5p, miR-338-5p, miR-375, and miR-548c-3p) were significantly increased in Hp-negative cancer tissues compared with Hp-positive cancer tissues. The expression of miRNAs in intestinal types of Hp infection-dependent GC suggests that there may be different GC pathogenesis between Hp-positive GC and Hp-negative GC. The findings of Yang and Song^39^ showed that the upregulation of RP11-169F17.1 and RP11-669N7.2 was significantly associated with worse overall survival and disease-free survival. RP11-169F17.1 and RP11-669N7.2 have strong associations with microRNAs in cancer, cell proliferation, and differentiation. RP11-169F17.1 and RP11-669N7.2 are closely related to gastritis, duodenal ulcer, GC, and mucosa-associated lymphoid tissue lymphoma caused by Hp infection. Therefore, RP11-169F17.1 and RP11-669N7.2 are new prognostic markers for gastric adenocarcinoma and may also play an important role in gastric diseases caused by Hp infection. These studies suggest that Hp virulence factors and genotypes have distinct effects on DEs.

Soyocak et al^40^ are the first to study the relationship of serum AhR, Zn, and B12 levels in the pathogenesis of Hp gastritis in adults. The study found that patients with Hp-positive CG had increased AhR levels, while Zn and B12 levels were decreased. AhR is a transcription factor that regulates genes related to toxicity metabolism and plays a role in antibacterial response as it does in other various mechanisms. The results suggest that bacterial infection caused by Hp can affect genes related to antibacterial mechanisms, thereby affecting the body’s absorption of micronutrients, leading to the occurrence of CG. Farah et al^41^ investigated the relationship between the presence of Hp infection and the platelet/lymphocyte ratio (PLR). Platelet/lymphocyte ratio was significantly elevated in Hp-infected patients compared with patients without Hp. Furthermore, patients with symptomatic Hp had higher PLRs than those with asymptomatic Hp. In addition, PLR increased with the severity of Hp symptoms. This study showed that Hp infection was significantly associated with PLR-based symptoms. The increase of PLR level in Hp patients aggravated the body inflammation and, to a certain extent, aggravated the occurrence of CG. Jia et al^42^ found that the expression of the lncRNA THAP9-AS1 was upregulated after infection of GC cells with Hp and was higher in GC tissues than in gastritis tissues. Colony formation, CCK8, and transwell assays were performed to show that THAP9-AS1 can promote GC cell proliferation and migration in vitro. The results of this study demonstrate that the lncRNA THAP9-AS1 is induced by Hp to promote GC cell growth and migration. These studies suggest that bacterial infection caused by Hp can affect genes related to antibacterial mechanisms and induce inflammation that may manifest as symptoms of CG.

Recently, through the statistical calculation and analysis of gene sequences and further experimental verification, we can further understand the relationship between certain genes and life activities. Some well-known RNAs have been widely used in diagnosing clinical tumors or as molecular targets and become faithful predictors of human health.^43,44^ HOTAIR is a known long non-coding RNA which has recently been associated with the progression of some cancer types. The overexpression of HOTATR promoted the proliferation and migration of GC cells, and the knockdown of HOTATR expression significantly inhibited tumor proliferation.^45^ The expression level of CCATI in GC tissues is significantly higher than that in healthy tissues, and its overexpression can promote the proliferation and invasion of tumor cells.^46^ GAS5 is an lncRNA with tumor-suppressing function, and its expression level is significantly downregulated in various tumors, such as breast and pancreatic cancers. Low GAS5 expression was significantly associated with low survival rate of GC and late Tumor Node Metastasis (TNM). The overexpression of GAS5 can promote GC cell proliferation and induce apoptosis. GAS5 interacts with the transcriptional activator YBX1 to affect the protein expression level of YBX1, thereby affecting the expression of p21 to induce cell cycle arrest.^47^ An increasing number of studies have found that RNAs are abnormally expressed in GC and contribute to suppressing or promoting cancer. In this study, we screened 11 important lncRNAs (AFAP1-AS1, MIR155HG, LINC00472, and FAM201A) and mRNAs (CASP10, SLC26A2, TRIB1, BMP2K, SCAMP1, TNKS1BP1, and MBOAT2) according to the gene expression profiles of gastric epithelial tissues in different stages of Hp infection-induced gastritis. By verifying the expression levels of key RNAs, it was confirmed that these key genes were significantly different between the healthy group and Hp-infected CG samples. This provides a target basis for the early diagnosis, control, and treatment of CG development after Hp infection.

## CONCLUSION

Based on the gene expression profile of gastric epithelial tissues at different stages of Hp infection-induced gastritis, key lncRNAs and genes in the development of gastritis induced by Hp infection were screened to provide a basis for early diagnosis and treatment of CG development after Hp infection. The samples from Bhutan and Dominican regions were subdivided into CG/normal comparison groups, and DERs were screened to obtain 1067 overlapping RNAs containing 21 lncRNAs and 1046 mRNAs. Through screening, 38 significant GO functional nodes, including 14 biological processes, 13 cellular components, and 11 molecular functions, were obtained, and 6 expression pattern clusters were obtained. Two ceRNA regulatory networks were constructed, and 4 shared miRNAs (i.e., hsa-miR-320b, hsa-miR-320c, hsa-miR-320d, and hsa-miR-155-5p) were obtained. Four miRNAs involved in the ceRNA regulatory network were extracted; 11 important lncRNAs and mRNAs regulated by these 4 miRNAs were obtained by screening. Cluster 1 had 6 lncRNAs and mRNAs (i.e., AFAP1-AS1, MIR155HG, TRIB1, BMP2K, SLC26A2, and CASP10), and cluster 2 contained 5 lncRNAs and mRNAs (i.e., LINC00472, FAM201A, SCAMP1, TNKS1BP1, and MBOAT2). This indicates that Hp infection has a certain influence on the development of gastritis. The 11 key RNAs obtained by screening can provide a target for the early diagnosis and treatment of CG after Hp infection.

## Figures and Tables

**Table 1.** Overlapping Genes Significantly Enrich the Information Table of Related GO Functional Nodes and KEGG Signaling Pathways

Category	Term	Count	Rich Factor	*P*	FDR
Biology process	GO:0006955~immune response	195	4.668046929	2.53E-81	4.61E-78
	GO:0006952~defense response	160	4.29728898	3.49E-60	6.36E-57
	GO:0006954~inflammatory response	109	5.539783977	4.22E-52	7.68E-49
	GO:0009611~response to wounding	129	4.020346949	1.64E-44	2.98E-41
	GO:0002684~positive regulation of immune system process	85	5.899180185	8.05E-43	1.47E-39
	GO:0001775~cell activation	88	5.064662012	2.16E-38	3.94E-35
	GO:0007242~intracellular signaling cascade	135	1.775390215	2.22E-11	4.05E-08
	GO:0007155~cell adhesion	87	2.052914704	1.56E-10	2.84E-07
	GO:0022610~biological adhesion	87	2.049986153	1.66E-10	3.03E-07
	GO:0043067~regulation of programmed cell death	95	1.932490061	5.22E-10	9.51E-07
	GO:0010941~regulation of cell death	95	1.9253766	6.20E-10	1.13E-06
	GO:0042981~regulation of apoptosis	92	1.890085592	3.13E-09	5.69E-06
	GO:0042127~regulation of cell proliferation	86	1.804984229	9.34E-08	1.70E-04
	GO:0007166~cell surface receptor linked signal transduction	157	1.397241169	6.66E-06	1.21E-02
Cellular component	GO:0005886~plasma membrane	359	1.553601114	1.25E-23	1.74E-20
	GO:0005887~integral to plasma membrane	148	2.036279246	7.67E-18	1.06E-14
	GO:0031226~intrinsic to plasma membrane	148	1.991028596	5.68E-17	1.55E-13
	GO:0044459~plasma membrane part	225	1.669398739	2.01E-16	3.11E-13
	GO:0009897~external side of plasma membrane	43	4.134391455	2.65E-15	3.69E-12
	GO:0009986~cell surface	59	2.771180586	1.79E-12	2.48E-09
	GO:0031224~intrinsic to membrane	427	1.272457551	1.03E-11	1.43E-08
	GO:0016021~integral to membrane	413	1.27441871	3.22E-11	4.46E-08
	GO:0044421~extracellular region part	111	1.889921675	4.51E-11	6.25E-08
	GO:0005615~extracellular space	87	2.075968413	7.92E-11	1.10E-07
	GO:0005576~extracellular region	184	1.496283289	4.22E-09	5.84E-06
	GO:0042613~MHC class II protein complex	12	6.763559397	5.17E-07	7.16E-04
	GO:0042611~MHC protein complex	14	4.014627361	2.98E-05	4.13E-02
Molecular function	GO:0019955~cytokine binding	36	5.59786332	2.47E-17	3.83E-14
	GO:0019956~chemokine binding	16	10.04390291	2.80E-12	4.35E-09
	GO:0005125~cytokine activity	34	2.95522528	3.50E-08	5.43E-05
	GO:0004896~cytokine receptor activity	17	5.23880845	6.16E-08	9.55E-05
	GO:0042379~chemokine receptor binding	15	5.188495764	5.28E-07	8.20E-04
	GO:0008528~peptide receptor activity, G-protein coupled	21	3.122200082	9.94E-06	1.54E-02
	GO:0001653~peptide receptor activity	21	3.122200082	9.94E-06	1.54E-02
	GO:0030246~carbohydrate binding	43	2.058787302	1.12E-05	1.74E-02
	GO:0030695~GTPase regulator activity	47	1.971799628	1.28E-05	1.98E-02
	GO:0060589~nucleoside-triphosphatase regulator activity	47	1.928830629	2.24E-05	3.48E-02
	GO:0042277~peptide binding	29	2.421298023	2.26E-05	3.50E-02
KEGG pathway	hsa04060:Cytokine-cytokine receptor interaction	71	3.479788341	6.19E-22	7.41E-19
	hsa04062:Chemokine signaling pathway	49	3.36473019	2.24E-14	2.68E-11
	hsa05330:Allograft rejection	20	7.133838384	1.36E-12	1.63E-09
	hsa04514:Cell adhesion molecules	34	3.307506887	7.09E-10	8.47E-07
	hsa04662:B cell receptor signaling pathway	24	4.109090909	4.36E-09	5.21E-06
	hsa04640:Hematopoietic cell lineage	24	3.583509514	7.75E-08	9.26E-05
	hsa04660:T cell receptor signaling pathway	27	3.210227273	1.12E-07	1.34E-04
	hsa04610:Complement and coagulation cascades	20	3.722002635	6.58E-07	7.86E-04
	hsa04650:Natural killer cell mediated cytotoxicity	29	2.799897471	7.28E-07	8.71E-04
	hsa04670:Leukocyte transendothelial migration	24	2.611710324	2.77E-05	3.31E-02
	hsa04666:Fc gamma R-mediated phagocytosis	21	2.838516746	2.91E-05	3.47E-02

GO, gene ontology; FDR, false discovery rate; MHC, major histocompatibility complex.

**Table 4.** The Significant Correlation between the Biological Processes and the KEGG Pathway of the Genes in Cluster1 ceRNA

Term	Count	*P*	Genes
GO:0031099~regeneration	2	.0297709	NOTCH1, SOCS3
GO:0006508~proteolysis	4	.0305087	CASP10, SOCS3, STAMBPL1, CTSC
GO:0032496~response to lipopolysaccharide	2	.0331153	SOCS3, TRIB1
GO:0002237~response to molecule of bacterial origin	2	.0368518	SOCS3, TRIB1
GO:0009611~response to wounding	3	.0389972	TNFRSF1B, NOTCH1, HPS5
GO:0048585~negative regulation of response to stimulus	2	.0426096	TNFRSF1B, SOCS3
hsa04920:Adipocytokine signaling pathway	2	.0320956	TNFRSF1B, SOCS3

**Table 5.** The Significant Correlation of Biological Process Associated with Genes in Cluster2 ceRNA

Term	Count	*P*	Genes
GO:0006955~immune response	3	.0089097	SARM1, ANXA11, EDA
GO:0044093~positive regulation of molecular function	2	.0357885	PAK1, EDA
GO:0006468~protein amino acid phosphorylation	2	.0396973	RIPK4, PAK1
GO:0007155~cell adhesion	2	.0412274	EDA, PDZD2
GO:0022610~biological adhesion	2	.0412732	EDA, PDZD2
GO:0016310~phosphorylation	2	.0456531	RIPK4, PAK1

**Table 2.** The Biological Processes of GO That Are Significantly Associated with mRNAs in Clusters 1 and 2

Category	Term	Count	*P*
Cluster1	GO:0006955~immune response	14	3.49E-04
	GO:0042981~regulation of apoptosis	15	4.51E-04
	GO:0009611~response to wounding	12	4.64E-04
	GO:0043067~regulation of programmed cell death	15	4.98E-04
	GO:0010941~regulation of cell death	15	5.17E-04
	GO:0043065~positive regulation of apoptosis	10	1.48E-03
	GO:0012501~programmed cell death	12	1.49E-03
	GO:0043068~positive regulation of programmed cell death	10	1.55E-03
	GO:0010942~positive regulation of cell death	10	1.60E-03
	GO:0007155~cell adhesion	12	4.27E-03
	GO:0022610~biological adhesion	12	4.32E-03
	GO:0006915~apoptosis	11	4.38E-03
	GO:0008219~cell death	12	5.22E-03
	GO:0010033~response to organic substance	12	5.33E-03
	GO:0016265~death	12	5.49E-03
	GO:0006952~defense response	10	1.49E-02
	GO:0042592~homeostatic process	11	1.90E-02
	GO:0042127~regulation of cell proliferation	11	2.54E-02
	GO:0007242~intracellular signaling cascade	14	4.92E-02
Cluster 2	GO:0009749~response to glucose stimulus	4	4.78E-03
	GO:0034284~response to monosaccharide stimulus	4	5.38E-03
	GO:0009746~response to hexose stimulus	4	5.38E-03
	GO:0009743~response to carbohydrate stimulus	4	1.04E-02
	GO:0051047~positive regulation of secretion	5	1.10E-02
	GO:0046883~regulation of hormone secretion	4	1.33E-02
	GO:0051046~regulation of secretion	6	2.39E-02
	GO:0046887~positive regulation of hormone secretion	3	2.40E-02
	GO:0048667~cell morphogenesis involved in neuron differentiation	6	2.70E-02
	GO:0048812~neuron projection morphogenesis	6	2.88E-02
	GO:0051050~positive regulation of transport	6	3.37E-02
	GO:0007264~small GTPase mediated signal transduction	7	3.91E-02
	GO:0007411~axon guidance	4	4.35E-02
	GO:0000904~cell morphogenesis involved in differentiation	6	4.53E-02
	GO:0048858~cell projection morphogenesis	6	4.59E-02
	GO:0060341~regulation of cellular localization	6	4.78E-02
	GO:0008610~lipid biosynthetic process	7	4.82E-02

GO, gene ontology.

**Table 3.** The KEGG Signaling Pathway Is Significantly Associated with mRNA in Clusters 1 and 2

Category	Term	Count	*P*
Cluster 1	hsa04662:B cell receptor signaling pathway	4	2.49E-03
	hsa04060:Cytokine-cytokine receptor interaction	6	6.82E-03
	hsa04210:Apoptosis	3	1.67E-02
	hsa04660:T cell receptor signaling pathway	3	2.32E-02
	hsa00260:Glycine, serine and threonine metabolism	2	2.32E-02
	hsa04623:Cytosolic DNA-sensing pathway	2	3.75E-02
	hsa04010:MAPK signaling pathway	4	3.95E-02
	hsa04621:NOD-like receptor signaling pathway	2	4.11E-02
	hsa04920:Adipocytokine signaling pathway	2	4.36E-02
	hsa04062:Chemokine signaling pathway	3	4.73E-02
	hsa04664:Fc epsilon RI signaling pathway	2	4.87E-02
Cluster 2	hsa00010:Glycolysis/Gluconeogenesis	3	9.45E-03
	hsa04080:Neuroactive ligand-receptor interaction	5	1.79E-02
	hsa00030:Pentose phosphate pathway	2	1.96E-02
	hsa04666:Fc gamma R-mediated phagocytosis	3	1.98E-02
	hsa00512:O-Glycan biosynthesis	2	2.30E-02
	hsa00051:Fructose and mannose metabolism	2	2.57E-02
	hsa04810:Regulation of actin cytoskeleton	4	2.85E-02
	hsa00562:Inositol phosphate metabolism	2	3.76E-02
	hsa04070:Phosphatidylinositol signaling system	2	4.77E-02
	hsa04520:Adherens junction	2	4.90E-02
